# Clinical Outcomes and Prognostic Factors for Salivary Duct Carcinoma: A Multi-Institutional Analysis of 141 Patients

**DOI:** 10.1245/s10434-015-5082-2

**Published:** 2016-01-20

**Authors:** Kuninori Otsuka, Yorihisa Imanishi, Yuichiro Tada, Daisuke Kawakita, Satoshi Kano, Kiyoaki Tsukahara, Akira Shimizu, Hiroyuki Ozawa, Kenji Okami, Akihiro Sakai, Yuichiro Sato, Yushi Ueki, Yukiko Sato, Toyoyuki Hanazawa, Hideaki Chazono, Kaoru Ogawa, Toshitaka Nagao

**Affiliations:** Department of Otorhinolaryngology-Head and Neck Surgery, Keio University School of Medicine, Tokyo, Japan; Department of Otorhinolaryngology, Saiseikai Yokohama Tobu Hospital, Yokohama, Japan; Department of Head and Neck Oncology and Surgery, International University of Health and Welfare Mita Hospital, Tokyo, Japan; Department of Otolaryngology-Head and Neck Surgery, Nagoya City University Graduate School of Medical Sciences, Nagoya, Japan; Department of Otolaryngology-Head and Neck Surgery, Hokkaido University Graduate School of Medicine, Sapporo, Japan; Department of Otolaryngology, Tokyo Medical University School of Medicine, Tokyo, Japan; Department of Otolaryngology-Head and Neck Surgery, Tokai University School of Medicine, Isehara, Japan; Department of Head and Neck Surgery, Niigata Cancer Center Hospital, Niigata, Japan; Department of Pathology, Cancer Institute Hospital, Japanese Foundation for Cancer Research, Tokyo, Japan; Department of Otolaryngology, Head and Neck Surgery, Chiba University Graduate School of Medicine, Chiba, Japan; Department of Anatomic Pathology, Tokyo Medical University School of Medicine, Tokyo, Japan

## Abstract

**Background:**

Among salivary gland malignancies, the prognosis of salivary duct carcinoma (SDC) is assumed to be the poorest. However, because of its low incidence, reliable survival estimates and prognostic factors based on a large number of patients remain to be elucidated, thereby making it impossible to standardize the optimal treatment for SDC.

**Methods:**

We performed this multi-institutional, retrospective analysis by collecting the clinical information of 141 patients with SDC without distant metastasis who underwent curative surgery as the initial treatment to elucidate overall survival (OS) and disease-free survival (DFS) along with their prognostic factors.

**Results:**

The 3-year OS and DFS rates were 70.5 and 38.2 %, respectively. Multivariate analysis revealed that age ≥65 years (*p* < 0.001) and N1 and N2 (*p* = 0.047 and <0.001, respectively) were independent prognostic factors for OS, whereas the primary site of the minor salivary and sublingual gland (*p* < 0.001) and N2 (*p* < 0.001) were those for DFS. The most common treatment failure was distant metastasis (55 patients, 39.0 %). For early parotid SDC, neither total parotidectomy in the patients with early T stage nor nerve resection in the patients without facial nerve palsy showed survival benefits.

**Conclusions:**

Advanced N stage independently affects both OS and DFS. Partial parotidectomy with facial nerve preservation could be a less invasive standard surgical procedure for parotid gland SDC in the early T stage without facial nerve palsy. Effective systemic therapy is imperative to improve DFS of SDC.

Salivary duct carcinoma (SDC), an aggressive and relatively rare tumor arising from the ductal epithelium of the salivary gland, represents approximately 10 % of all salivary gland malignancies.^1^,^2^ Although SDC was first reported by Kleinsasser in 1968, it was officially defined as a distinctive clinicopathologic entity in the revised histologic classification of salivary gland neoplasms by the World Health Organization in 1990.^3^,^4^ SDC morphologically resembles ductal carcinoma of the breast while considering the histological features, such as ductal formation with a solid, cystic, cribriform, or papillary structure; elements of intraductal comedonecrosis; calcification; and a reactive desmoplastic stroma.^1^,^2^,^5^,^6^ In general, SDC tends to be diagnosed in men in their 60s or 70s predominantly in the parotid gland, which is the most common primary site.^1^,^2^,^7^,^8^ Clinically, SDC is characterized by aggressive behavior with a tendency for rapid progression, including early facial nerve involvement, extra-glandular invasion, and high incidence of regional and distant metastasis, leading to tumor-related death.^7^–^10^

Similar to other high-grade salivary gland tumors, the standard treatment for resectable tumors is radical surgery, including ipsilateral neck dissection, followed by postoperative adjuvant radiotherapy. Unfortunately, because of the high incidence of locoregional recurrence and distant metastases, the latter reportedly being 40–70 %, most patients die of the disease within 3 years. Hence, the prognosis of SDC appears to be one of the poorest among salivary gland cancers.^6^,^8^–^13^ However, due to its very low incidence, these previous studies on clinical outcomes with survival analysis have been performed in a small number of patients, ranging from 13 to 56. Thus, reliable survival estimates and prognostic factors of SDC based on a large number of patients remain to be clarified, except for a recent study that analyzed the data from the Surveillance, Epidemiology and End Results (SEER) program of the National Cancer Institute.^14^ For the same reason, it remains impossible to conduct large-scale clinical trials to discover a promising chemotherapy protocol for patients with SDC or other salivary gland malignancies.

To overcome the inevitable limitations of single-institute clinical investigations, we organized a multi-institutional research group to collect the clinical and histopathological information from a large number of patients with SDC. We performed this retrospective analysis to elucidate the clinical prognostic factors along with the clinical outcomes of the patients with SDC treated with curative intent at seven tertiary hospitals.

## Methods

### Patients and Treatments

The present study was approved by the Institutional Ethics Review Board of each of the seven institutions that participated in the study; the requirement for obtaining informed consent was waived owing to the retrospective nature of the analysis. The clinical data of 186 patients with SDC diagnosed at those institutions between 1992 and 2014 were obtained, of which 141 patients without distant metastasis at initial diagnosis who underwent curative surgery as the initial treatment were enrolled in this study. All patients underwent central pathological review by two expert pathologists (T.N. and Y.S.) and were staged according to the UICC TNM classification and staging system (2010, 7th edition).^15^

The surgical procedure for the primary tumor was determined according to the tumor site and its extent, which were precisely evaluated by imaging diagnosis using CT, MRI, US, and/or PET-CT. Neck dissection was performed for patients with lymph node metastasis (N1 and N2 patients) and/or those (including N0 patients) with histopathological diagnosis of high-grade malignancy indicated preoperatively by aspiration cytology. Postoperative radiotherapy of 60–66 Gy was given when any of the resection margins was positive or equivocal and/or lymph node metastasis was pathologically positive. In cases of grossly positive margins and/or extracapsular spread, concurrent chemoradiotherapy or adjuvant chemotherapy was administered at the surgeon’s discretion.

### Statistical Analysis

Overall survival (OS) and disease-free survival (DFS) rates were estimated using the Kaplan–Meier method. The prognostic effects of patient/disease factors, including age, sex, primary site, tumor size, facial nerve palsy, N classification, rapid tumor progression (defined as a rapid growth of the primary tumor as observed by the patient immediately prior to visiting a clinic or hospital), and pain (defined as a pain that the patient had in the lesion), were first examined by univariate analysis using the log-rank test and the Cox’s proportional hazards model; the latter further assessed the independent significance of these factors on multivariate analysis without sequential and/or stepwise variable selection. *p* values <0.05 were considered statistically significant. All statistical analyses were performed using STATA ver. 13 (StataCorp., TX).

We also investigated the patterns of treatment failure, including locoregional recurrence and distant metastasis. To evaluate the possible validity of less invasive procedures for SDC of the parotid gland in the early T stage and/or without facial nerve palsy, we examined differences in the clinical outcomes including locoregional control (LRC) rate between patients who underwent partial parotidectomy and total parotidectomy, as well as those between nerve preservation and nerve resection.

## Results

### Patient Characteristics

The clinical and demographic characteristics of the 141 patients that comprised 119 men (84.4 %) and 22 women (15.6 %) are summarized in Table 1. The median age at initial diagnosis was 64 (range 26–85) years. The primary tumor site was the parotid gland in 112 patients (79.4 %), submandibular gland in 25 (17.7 %), minor salivary gland in 3 (2.1 %), and sublingual gland in 1 (0.7 %). Approximately two-thirds of the patients (93 patients, 66.0 %) presented with a T3/T4 tumor. Lymph node metastasis was clinically positive (N1/N2) in more than a half of the patients (71 patients, 50.4 %). Among the 138 patients with primary tumor size information, 85 patients had tumors <40 mm in size and 53 patients had tumors ≥40 mm. Forty-three patients (30.5 %) presented with facial nerve palsy, all of whom had primary tumors at the parotid gland. Thirty-four patients (24.1 %) had a recent history of rapid progression of the tumor, and 25 patients (17.7 %) experienced pain due to the tumor.

**Table 1:** Patient characteristics (*n* = 141)

	*N*	(%)
Age		
<65	78	(55.3)
≥65	63	(44.7)
Sex		
Men	119	(84.4)
Women	22	(15.6)
Primary tumor site		
Parotid gland	112	(79.4)
Submandibular gland	25	(17.7)
Minor salivary gland	3	(2.1)
Sublingual gland	1	(0.7)
Tumor size		
<40 mm	85	(60.3)
≥40 mm	53	(37.6)
Unknown	3	(2.1)
Facial nerve palsy		
−	96	(68.1)
+	43	(30.5)
Unknown	2	(1.4)
N classification		
0	70	(49.6)
1	9	(6.4)
2	62	(44.0)
Rapid progression		
−	101	(71.6)
+	34	(24.1)
Unknown	6	(4.3)
Pain		
−	112	(79.4)
+	25	(17.7)
Unknown	4	(2.8)
PORT		
−	58	(41.1)
RT	58	(41.1)
CRT	25	(17.7)

*PORT* post-operative radiotherapy, *RT* radiotherapy, *CRT* chemoradiation

While 83 patients underwent surgery followed by adjuvant radiotherapy or chemoradiotherapy, 51 patients underwent surgery alone, and the remaining 7 patients received adjuvant chemotherapy without radiotherapy. Surgical procedures were extended total parotidectomy (composite resection of the parotid gland along with surrounding structures) in 31 patients, total parotidectomy in 60, partial parotidectomy (lobectomy) in 19, parapharyngeal tumorectomy in 4, submandibular gland resection in 25, and partial maxillectomy and extended sublingual gland resection in 1 each.

### Clinical Outcome and Survival Analysis

**Fig. 1:**
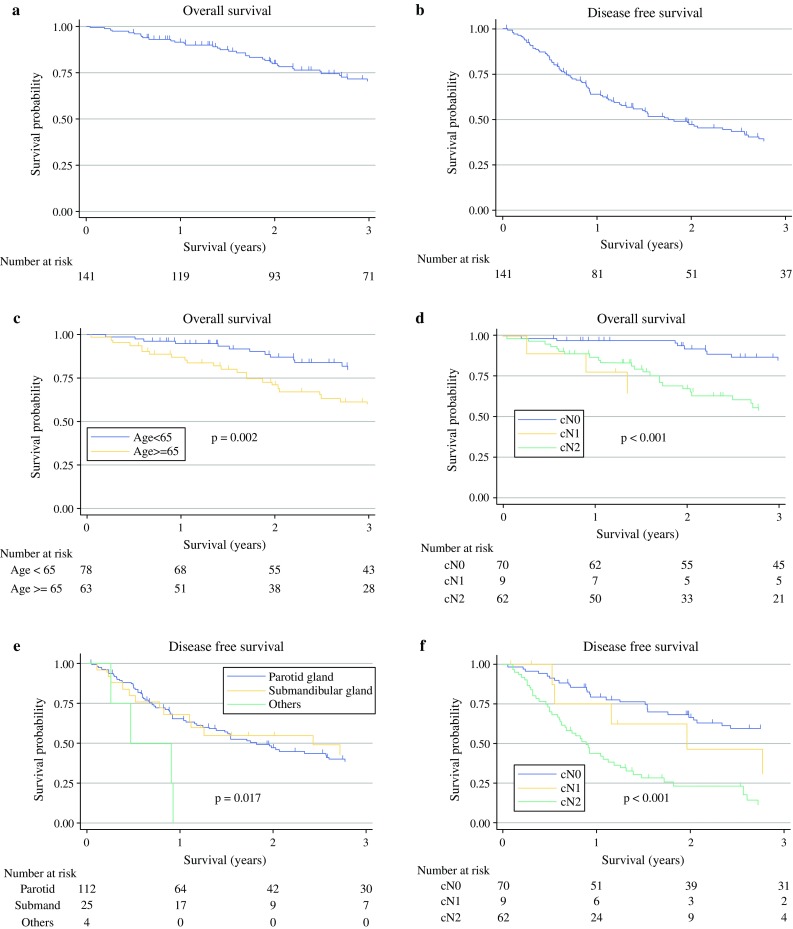
Actuarial survival curves of patients with SDC. **a** Overall survival (OS) and **b** disease-free survival (DFS) of all 141 patients with SDC. The 3-year OS and DFS rates were 70.5 and 38.2 %, respectively. The survival curves according to each of the prognostic factors that were found to be significant on both univariate analysis with the log-rank test and multivariate analysis with Cox’s hazards model are shown as follows: **c**, **d** OS according to age (*p* = 0.002) and N classification (shown as cN, *p* < 0.001), respectively. **e**, **f** DFS according to primary tumor site (*p* = 0.017) and N classification (shown as cN, *p* < 0.001), respectively

The median follow-up period was 36 months. At the time of analysis, 55 patients were alive without the disease (including 7 patients who underwent secondary or tertiary salvage treatment and remained recurrence-free), 44 died of disease recurrences (2 of local, 1 of local and distant, 2 of regional, 7 of regional and distant, 25 of distant, 1 of treatment-related, and 6 unspecified), 29 patients were alive with the disease, and 13 died of other causes. The 3-year OS and DFS rates were 70.5 % [95 % confidence interval (CI) 61.4–77.8 %] and 38.2 % (95 % CI 29.5–46.9 %), respectively (Fig. 1a, b).

The results of the univariate analysis for the prognostic factors determined by log-rank tests are displayed, in part, in Fig. 1c–f, whereas those analyzed by Cox’s hazards model are summarized in Table 2. The OS was significantly worse in patients aged ≥65 years (*p* = 0.002, vs. <65 years), those with N1 and N2 (*p* = 0.036 and <0.001, respectively, vs. N0), and those showing rapid progression (*p* = 0.003), whereas no significant difference was found regarding other factors. The DFS was significantly worse when primary tumors were at the minor salivary gland and sublingual gland (*p* = 0.009, vs. the parotid gland), in patients with N2 (*p* < 0.001, vs. N0), those showing rapid progression (*p* = 0.014), and those with pain (*p* = 0.032), whereas other factors showed no significant difference. Multivariate analysis revealed that age ≥65 years [hazard ratio (HR) = 2.96, *p* < 0.001, vs. <65 years] and N1 and N2 (HR = 2.97 and 4.01, *p* = 0.047 and <0.001, respectively, vs. N0) were independent prognostic factors for OS, while the primary sites of the minor salivary gland and sublingual gland (HR = 8.46, *p* < 0.001, vs. the parotid gland) and N2 (HR = 3.94, *p* < 0.001, vs. N0) were independent prognostic factors for DFS (Table 2).

**Table 2:** Univariate and multivariate analysis for overall survival and disease-free survival (*n* = 141)

Variables	*N*	Overall survival						Disease-free survival					
		Univariate analysis			Multivariate analysis			Univariate analysis			Multivariate analysis		
		HR	95 % CI	*p* value	HR	95 % CI	*p* value	HR	95 % CI	*p* value	HR	95 % CI	*p* value
Age													
<65	78	1.00	–	–	1.00	–	–	1.00	–	–	1.00	–	–
≥65	63	2.33	1.36–3.99	0.002*	2.96	1.62–5.41	<0.001*	1.20	0.78–1.86	0.405	1.62	0.98–2.68	0.058
Sex													
Men	119	1.00	–	–	1.00	–	–	1.00	–	–	1.00	–	–
Women	22	0.96	0.47–1.97	0.916	1.38	0.60–3.19	0.448	0.69	0.36–1.34	0.276	0.71	0.34–1.49	0.370
Primary tumor site													
Parotid gland	112	1.00	–	–	1.00	–	–	1.00	–	–	1.00	–	–
Submandibular gland	25	1.17	0.60–2.27	0.648	1.37	0.59–3.16	0.459	1.03	0.59–1.82	0.906	1.48	0.72–3.05	0.284
Others	4	1.54	0.37–6.39	0.551	1.54	0.29–8.17	0.613	3.98	1.42–11.15	0.009*	8.46	2.61–27.45	<0.001*
Tumor size													
<40 mm	85	1.00	–	–	1.00	–	–	1.00	–	–	1.00	–	–
≥40 mm	53	1.32	0.76–2.33	0.324	1.03	0.54–1.96	0.931	1.36	0.86–2.13	0.185	1.29	0.77–2.16	0.339
Unknown	3	–	–	–	–	–	–	–	–	–	–	–	–
Facial nerve palsy													
−	96	1.00	–	–	1.00	–	–	1.00	–	–	1.00	–	–
+	43	1.39	0.80–2.40	0.239	1.50	0.77–2.94	0.233	1.49	0.95–2.35	0.085	1.69	0.99–2.88	0.054
Unknown	2	–	–	–	–	–	–	–	–	–	–	–	–
N classification													
0	70	1.00	–	–	1.00	–	–	1.00	–	–	1.00	–	–
1	9	2.70	1.07–6.80	0.036*	2.97	1.02–8.70	0.047*	2.12	0.91–5.36	0.078	1.92	0.71–5.18	0.195
2	62	3.29	1.83–5.91	<0.001*	4.01	2.04–7.90	<0.001*	4.05	2.51–6.55	<0.001*	3.94	2.34–6.63	<0.001*
Rapid progression													
−	101	1.00	–	–	1.00	–	–	1.00	–	–	1.00	–	–
+	34	2.40	1.36–4.24	0.003*	1.73	0.87–3.42	0.116	1.82	1.13–2.93	0.014*	1.18	0.68–2.07	0.556
Unknown	6	–	–	–	–	–	–	–	–	–	–	–	–
Pain													
−	112	1.00	–	–	1.00	–	–	1.00	–	–	1.00	–	–
+	25	1.51	0.82–2.78	0.182	1.34	0.63–2.84	0.451	1.74	1.05–2.89	0.032*	1.78	0.94–3.35	0.076
Unknown	4	–	–	–	–	–	–	–	–	–	–	–	–

*HR* hazard ratio, *CI* confidence interval

* Statistically significant (*p* < 0.05)

### Patterns of Treatment Failure

**Fig. 2:**
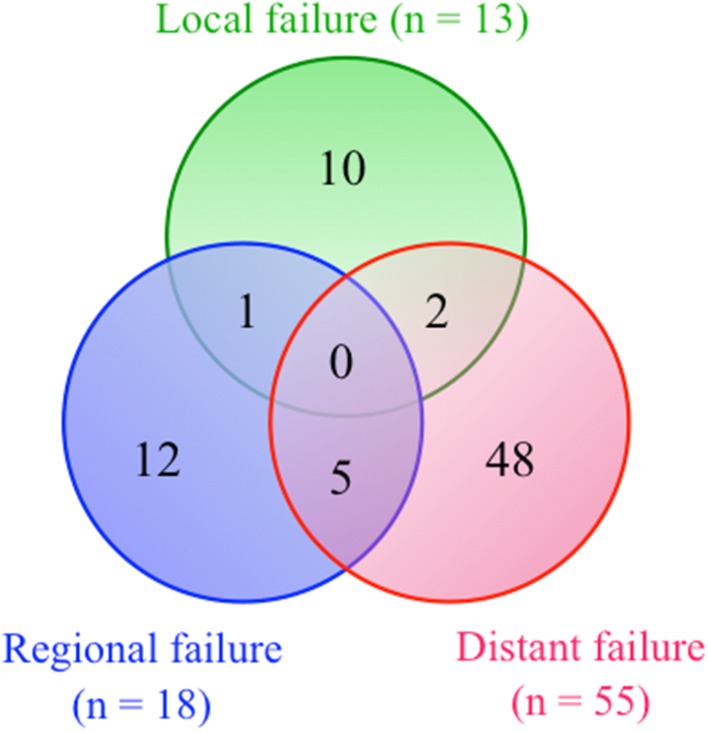
Distribution of treatment failure patterns in 141 patients with SDC. Thirteen cases (9.2 %) of local, 18 cases (12.8 %) of regional, and 55 cases (39.0 %) of distant failures were observed in a total of 78 patients

As shown in Fig. 2, treatment failure occurred in 78 patients (55.3 %), including 13 (9.2 %) local, 18 (12.8 %) regional, and 55 (39.0 %) distant failures, of which 48 were without locoregional failure. The most common sites of distant metastasis were the lungs (*n* = 32), followed by the bones (*n* = 11), liver (*n* = 5), and brain (*n* = 3).

### Partial Parotidectomy vs. Total Parotidectomy for Parotid SDC in the Early T Stage

The clinical outcomes of 33 patients with SDC of T1-2 of the parotid gland who underwent partial parotidectomy or total parotidectomy were compared (Table 3a). Univariate analysis, as well as multivariate analysis adjusted for age, sex, tumor size, N classification, rapid progression, pain, and adjuvant radiotherapy, showed no significant difference in OS, DFS, and LRC.

**Table 3:** The impact of total parotidectomy in early T stage SDC (*n* = 33) and facial nerve resection in facial nerve palsy-negative SDC (*n* = 68) of the parotid gland on clinical outcome

(a) Early T stage SDC of the parotid gland (*n* = 33)								
Endpoint	Procedure	*N*	Univariate analysis			Multivariate analysis^a^		
	Extent of parotidectomy		HR	95 % CI	*p* value	HR	95 % CI	*p* value
Overall survival	Partial	15	1.00	–	–	1.00	–	–
	Total	18	1.24	0.29–5.21	0.771	not calculable	–	–
Disease-free survival	Partial	15	1.00	–	–	1.00	–	–
	Total	18	1.60	0.48–5.32	0.443	0.18	0.01–2.83	0.225
Locoregional control	Partial	15	1.00	–	–	1.00	–	–
	Total	18	2.16	0.22–20.90	0.507	not calculable	–	–

(b) Facial nerve palsy-negative SDC of the parotid gland (*n* = 68)								
Endpoint	Procedure	*N*	Univariate analysis			Multivariate analysis^a^		
	Facial nerve		HR	95 % CI	*p* value	HR	95 % CI	*p* value
Overall survival	Preservation	27	1.00	–	–	1.00	–	–
	Resection	41	2.92	1.08–7.90	0.035*	0.91	0.23–3.53	0.890
Disease-free survival	Preservation	27	1.00	–	–	1.00	–	–
	Resection	41	3.54	1.54–8.16	0.003*	2.10	0.71–6.21	0.179
Locoregional control	Preservation	27	1.00	–	–	1.00	–	–
	Resection	41	4.57	1.02–20.47	0.047*	1.63	0.26–10.23	0.604

*HR* hazard ratio, *CI* confidence interval

* Statistically significant (*p* < 0.05)

^a^Adjusted by age, sex, tumor size, *N* classification, rapid progression, pain, and adjuvant radiotherapy

### Nerve Preservation vs. Nerve Resection for Parotid SDC Without Facial Nerve Palsy

The clinical outcomes of 68 patients with SDC without facial nerve palsy who underwent surgery with nerve preservation or nerve resection were compared (Table 3b). Univariate analysis showed that OS, DFS, and LRC of the patients who underwent facial nerve resection were significantly worse than those whose facial nerves were preserved. However, no significant difference was found in OS, DFS, and LRC, on multivariate analysis adjusted as described above.

## Discussion

There has been an absence of studies analyzing the survival and prognostic factors of SDC based on a large number of patients because of its low incidence. To the best of our knowledge, the present study analyzed the largest series of patients with SDC, except for a U.S. study using the SEER data in which only disease-specific survival (DSS) was described.^14^ Although the retrospective nature of the study inevitably could not exclude selection bias, especially for additional treatment after surgery, the results obtained from our study could provide reliable survival estimates and prognostic factors for patients with SDC.

Two early studies with small cohorts (*n* = 26 each) in the 1990s reported considerably poor outcomes of 2-year OS of 42.3 and 58 %, and 5-year OS of 11.5 and 30 %, respectively.^8^,^10^ Although a later study with a larger cohort (*n* = 59) also showed a comparable outcome of the 2-, 3-, and 5-year OS of 62.3, 42.7, and 26.9 %, respectively, more recent studies with similar cohort sizes reported somewhat better 5-year OS outcomes of 55.1 % (*n* = 35), 42 % (*n* = 56), and 43 % (*n* = 54), suggesting the benefit of intensification of both surgery and adjuvant radiotherapy regarding the treatment outcomes.^12^,^13^,^16^,^17^ However, there were noticeably large differences between OS and DFS, i.e., the 5-year DFS was 29 %.^12^ In our study, the 3-year OS and DFS were 70.5 and 38.2 %, respectively. This reflected a considerably high ratio of treatment failure (55.3 % in our study) for this disease.

Multivariate analysis in the present study showed, for the first time, that advanced N stage independently affects both OS and DFS in patients with SDC. Significant correlation of the N classification with OS has been previously reported, although those results were based on only univariate analyses with much smaller sample sizes.^8^,^10^,^16^ Our findings also were partially consistent with those of the SEER study (*n* = 228), in which multivariate analysis showed that age and N classification, as well as tumor size and grade, were independent prognostic factors for OS and DSS (DFS was not analyzed).^14^ Although the influence of age on prognosis may depend on the cancer type, the prognostic significance revealed in the OS of patients with SDC may reflect an increased risk of death in the elderly owing to other fatal diseases generally associated with aging.

In an early study of 30 patients with SDC that included 13 patients with distant metastasis at initial diagnosis, multivariate analysis showed that tumor diameter and distant metastasis were independent prognostic factors for OS.^18^ Given that some subjects had distant metastasis, unlike most other studies, distant metastasis could inevitably be the most unfavorable predictor of survival unless an effective systemic therapy becomes available for such patients. In another recent study that employed multivariate analysis, lymphovascular invasion and perineural invasion were found to be independent histopathological prognostic factors for OS, suggesting a possible benefit of evaluating histopathological characteristics for predicting survival of patients with SDC.^17^

In our cohort, the most common form of treatment failure was distant metastasis, which corroborates the findings of a limited number of previous observations in smaller cohorts.^13^,^17^ Consistent with our findings, the lungs and bones were the most common sites of distant metastasis in SDC.^11^,^13^,^17^ Such a high ratio of distant metastasis is presumed to be the leading cause of low DFS. Although extended resection with wider margins combined with intensified adjuvant radiotherapy have seemingly contributed to better treatment outcomes of SDC by improving LRC, these strategies alone cannot prevent the development of delayed distant metastasis. Therefore, an effective systemic therapy after curative surgery is imperative to improve DFS of SDC patients. Unfortunately, there has been no evidence-based chemotherapy regimen for salivary gland cancers including SDC.^19^ Recent immunohistochemical studies reported that androgen receptor (AR) expression is observed in 43–92 % of SDC, whereas HER2 expression is observed in 26–77 %, both of which were confirmed in our separated subanalysis (data not shown), suggesting a potential role for agents acting on these receptors as possible molecular-targeted therapy for SDC.^16^,^20^–^31^ Currently, our multi-institutional joint research group is conducting interventional clinical studies of systemic therapy targeting AR and HER2 in combination with chemotherapy for recurrent/metastatic SDC.

Although preservation of the facial nerve has been recommended during parotid gland cancer surgery if nerve function was preoperatively normal, its applicability for SDC remains to be determined.^32^,^33^ In our subanalyses regarding patients with early SDC of the parotid gland, neither total parotidectomy in patients with early T stage nor nerve resection in patients without facial nerve palsy showed a survival benefit compared with those treated with partial parotidectomy and nerve preservation, respectively. Although these results should be interpreted while considering the possible selection bias because of the retrospective nature of the study, our results suggest that even in SDC of the parotid gland, partial parotidectomy with facial nerve preservation could be the standard procedure for primary tumors in early T stage without facial nerve palsy.

## Conclusions

Our multi-institutional joint study revealed that advanced N stage independently affects both OS and DFS. Given the high incidence of distant failure, an effective systemic therapy is essential for improving DFS of SDC. Partial parotidectomy with facial nerve preservation may constitute a less invasive standard surgical procedure for parotid gland SDC in the early T stage without facial nerve palsy.
